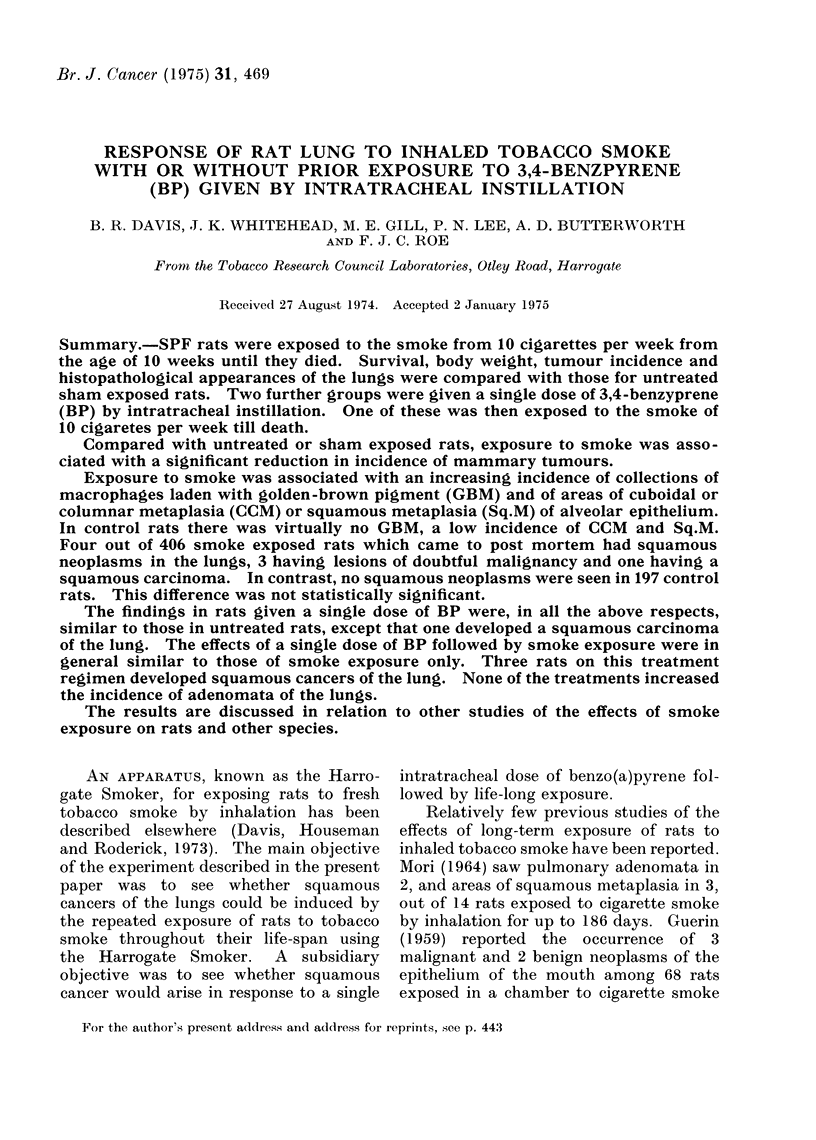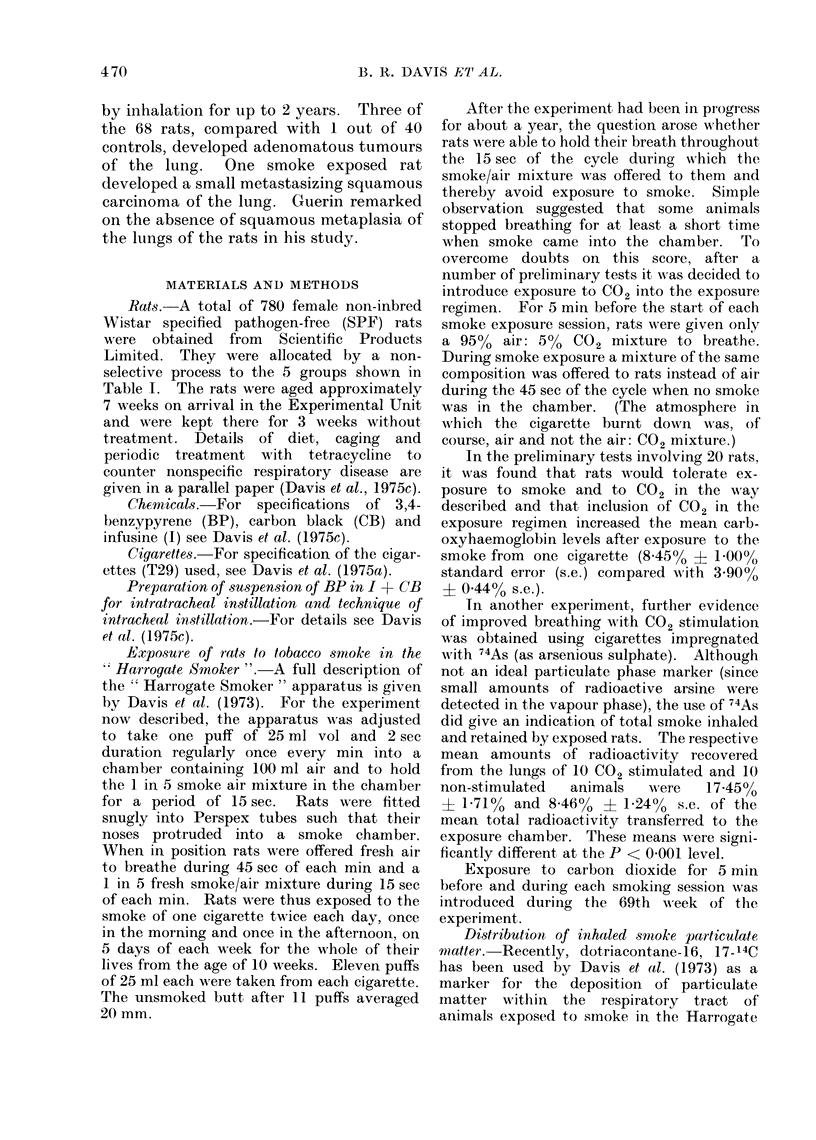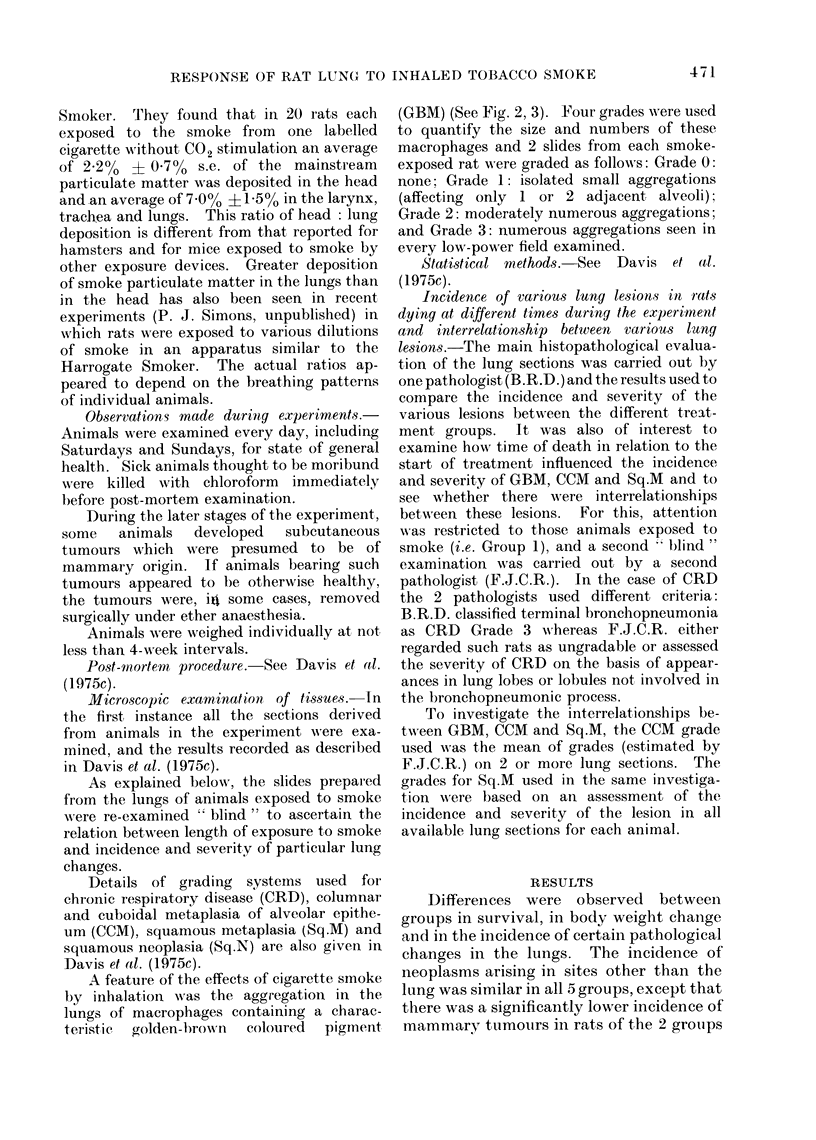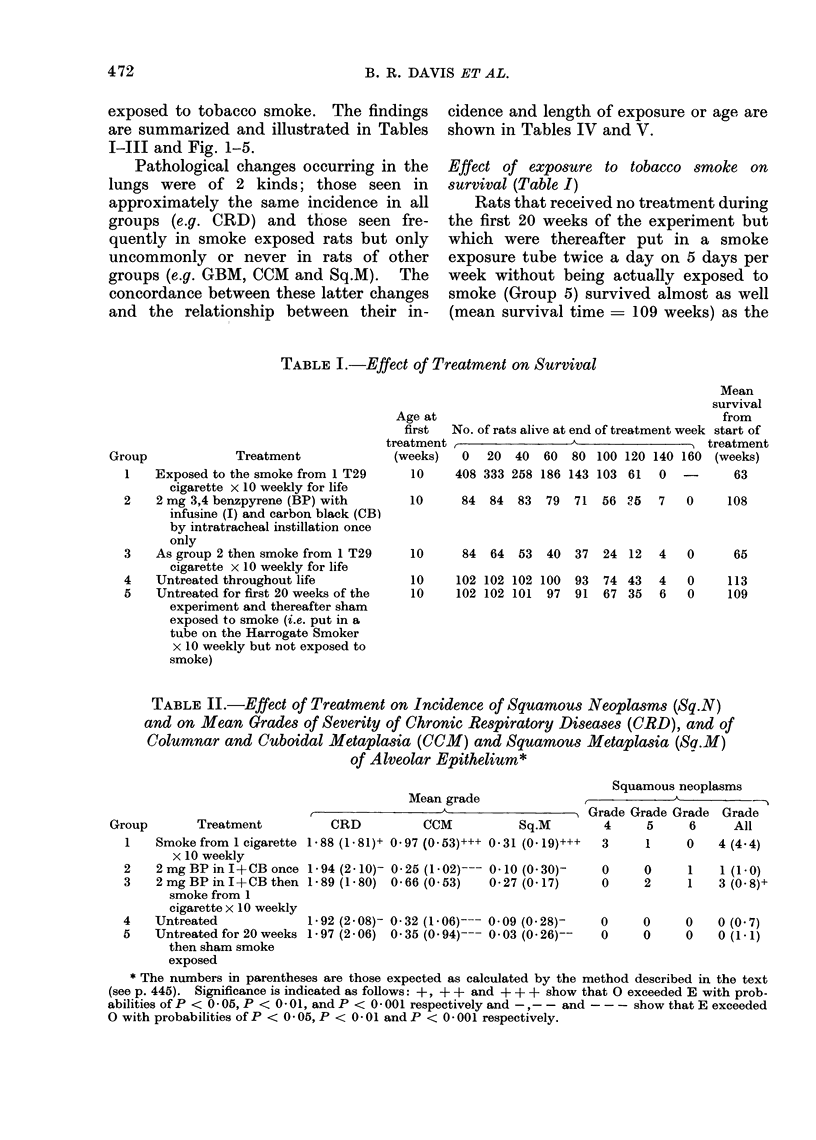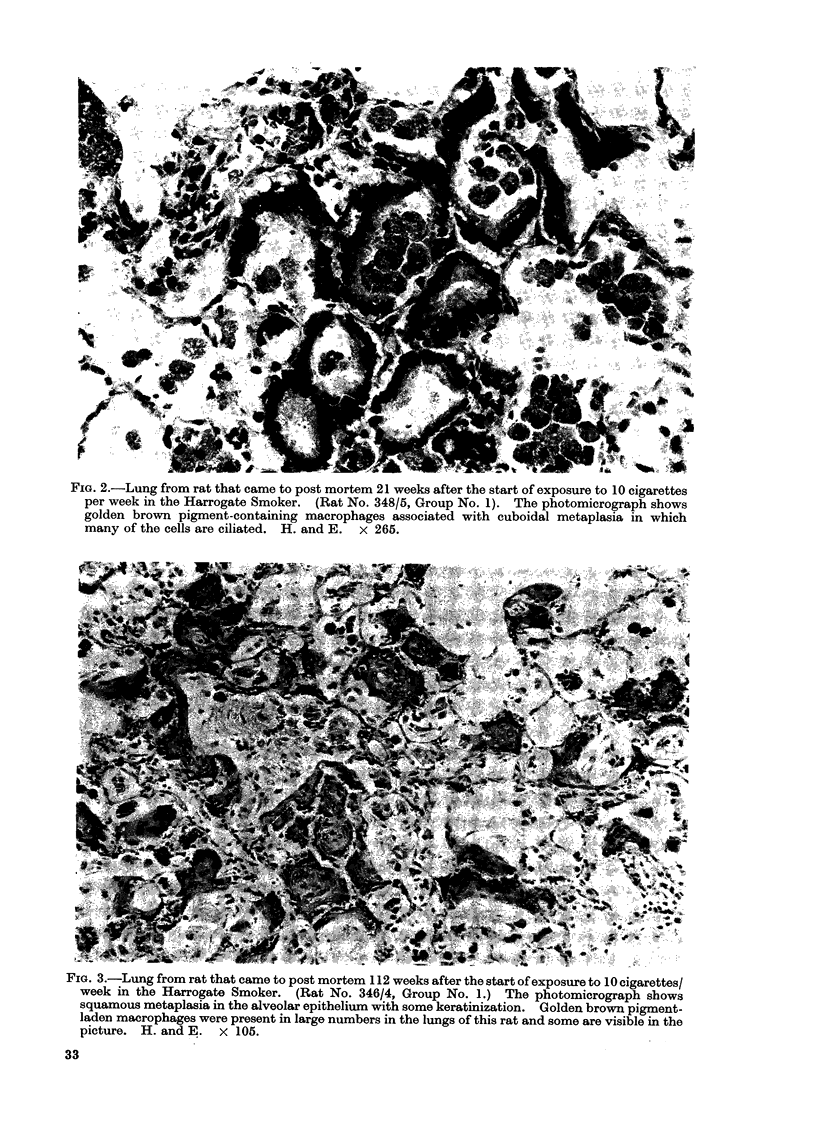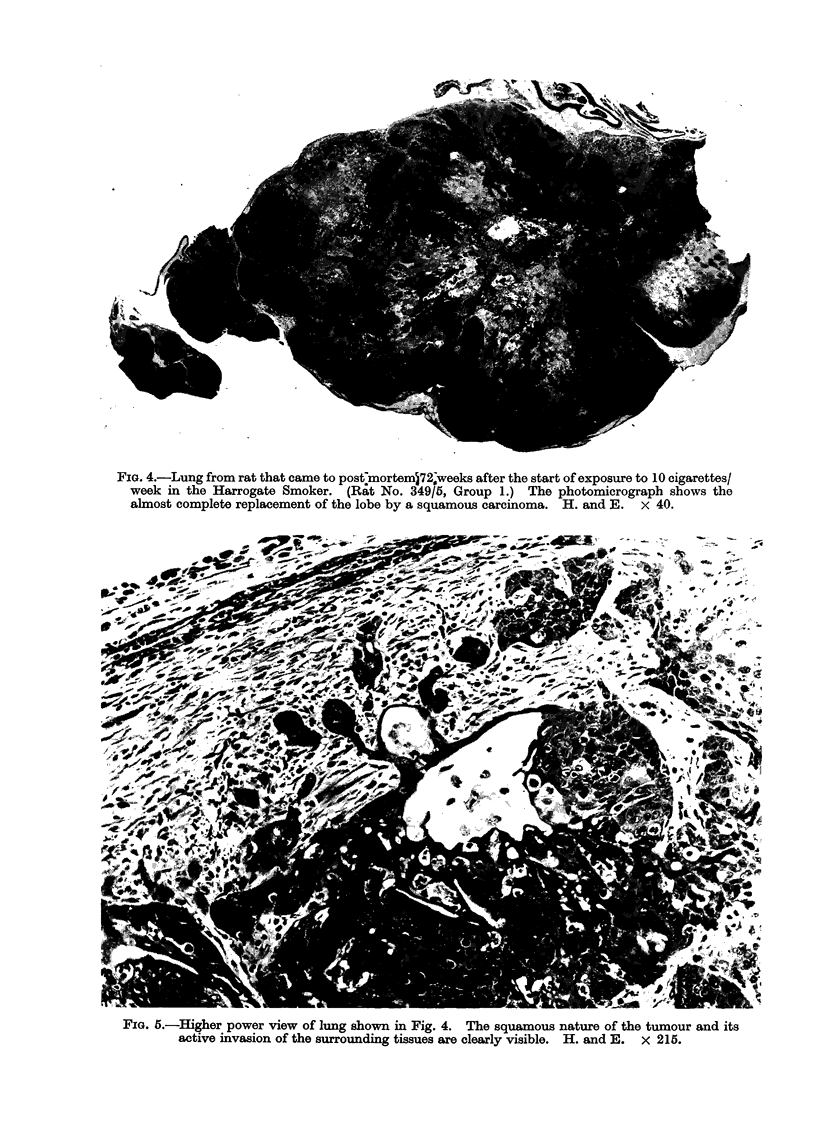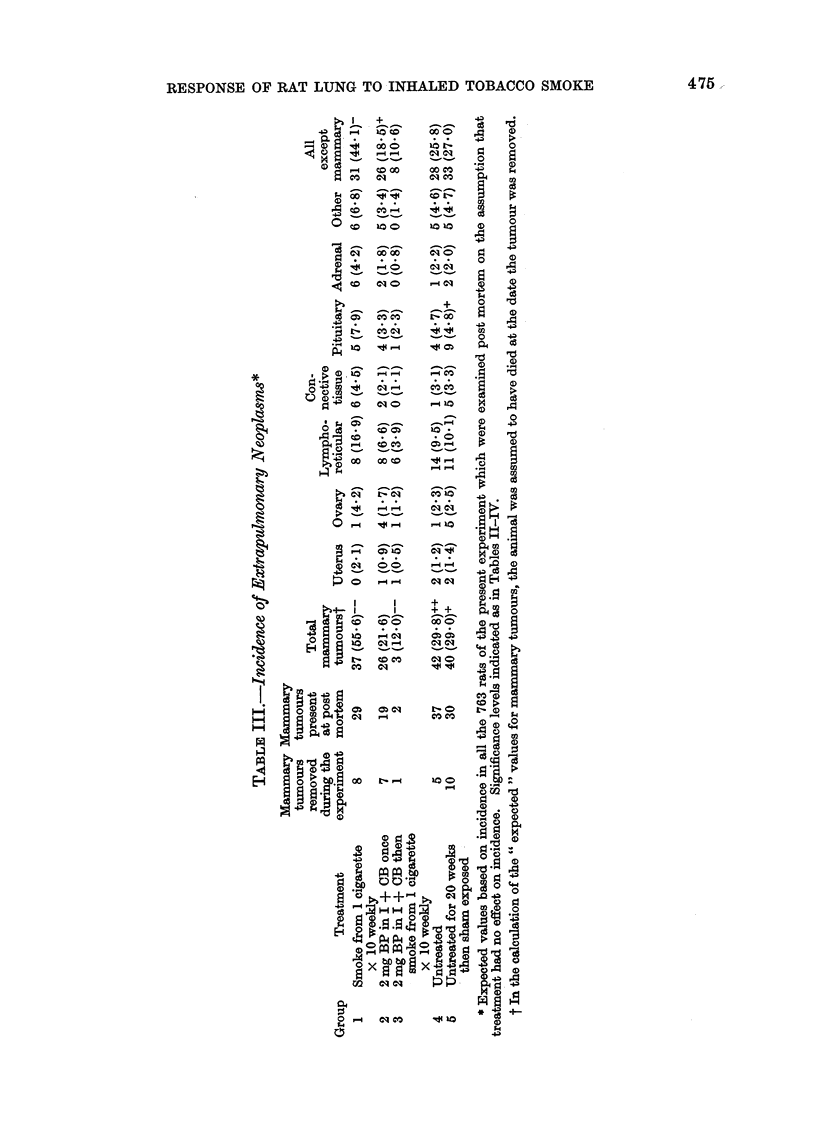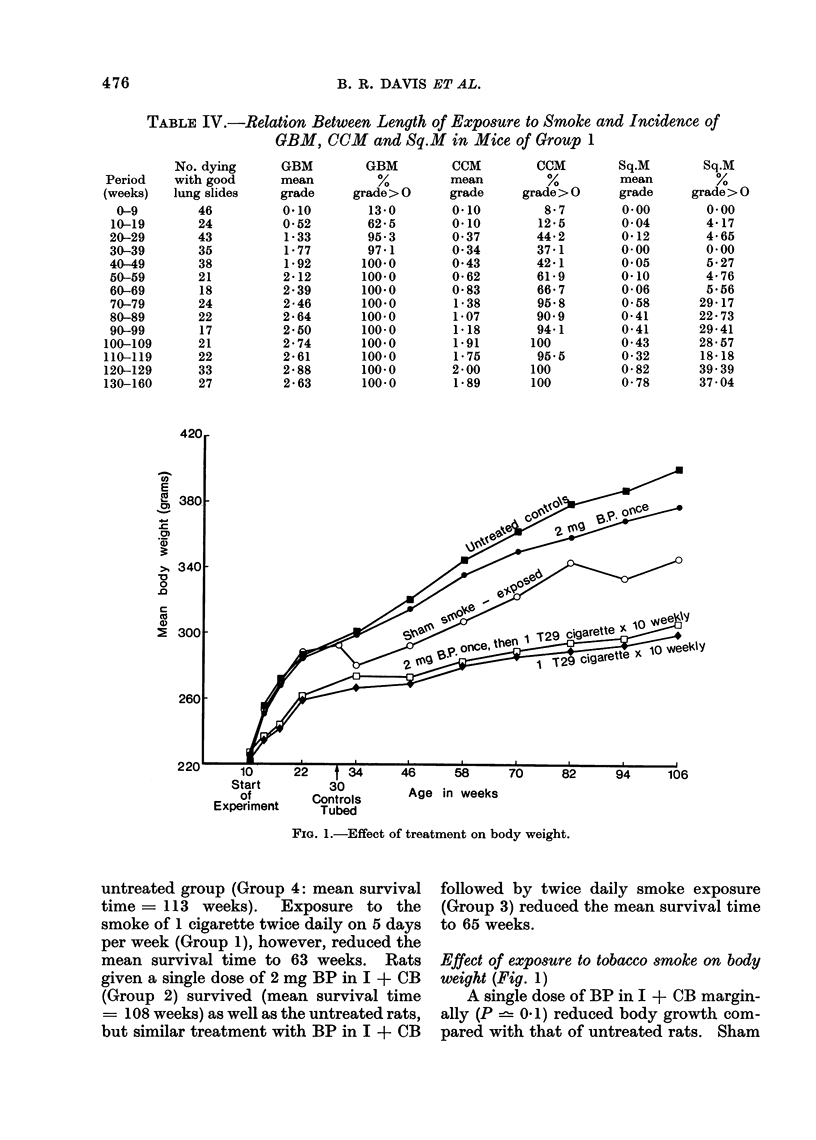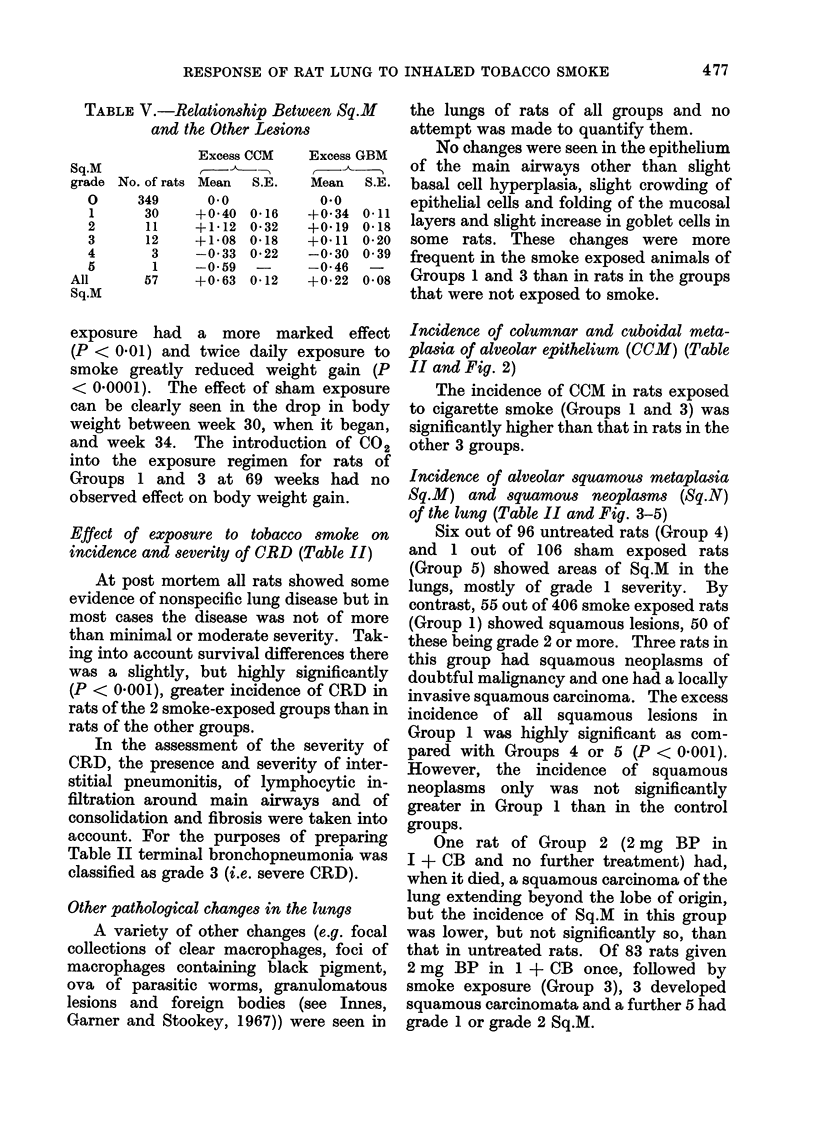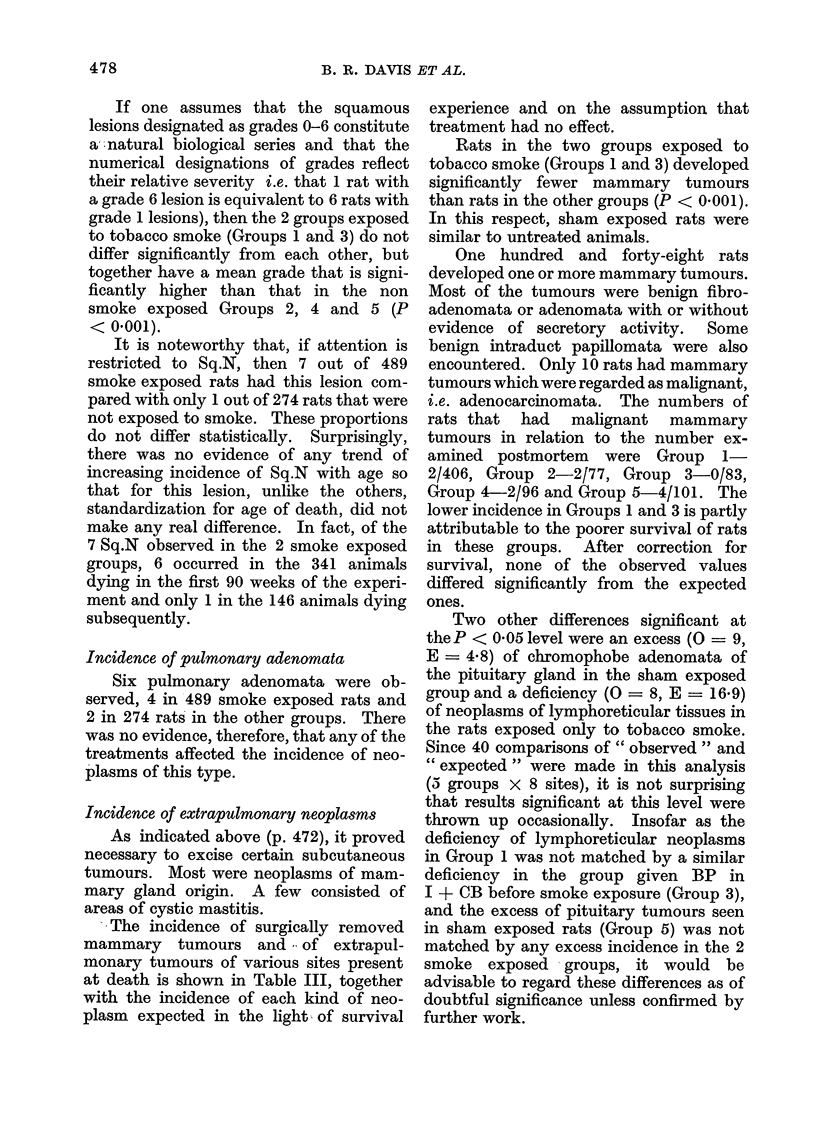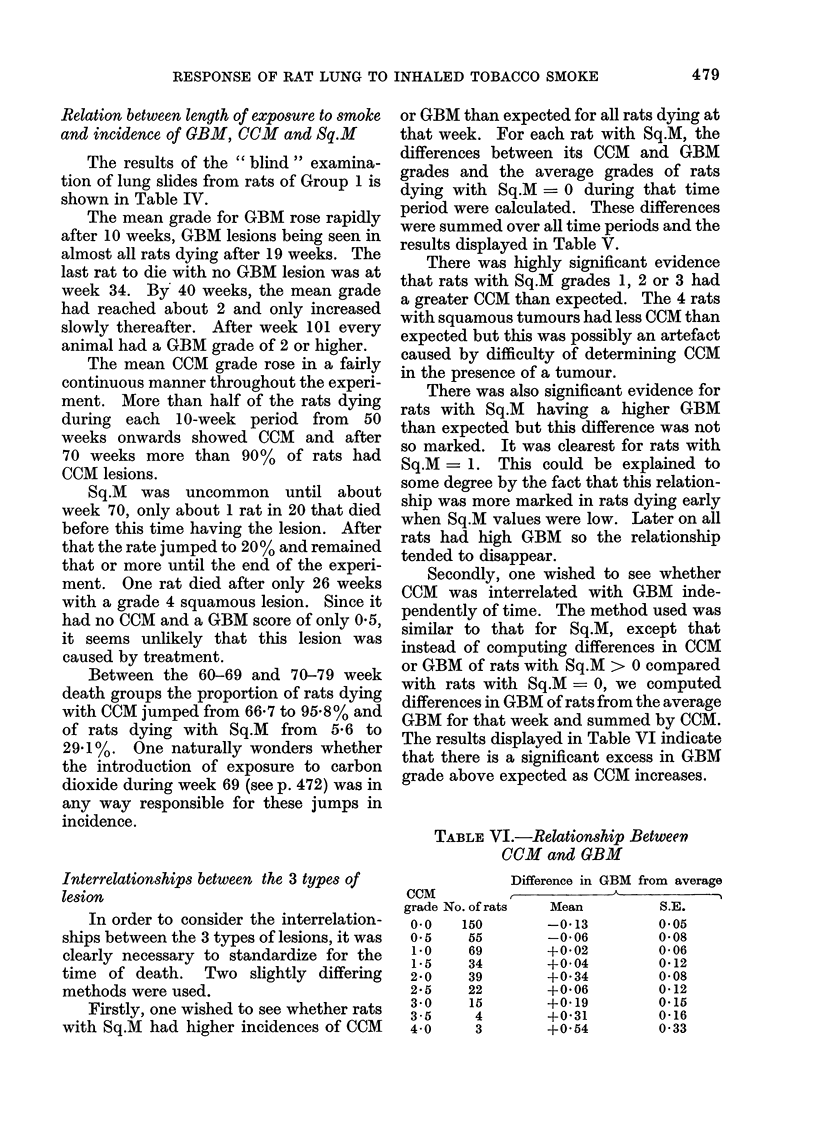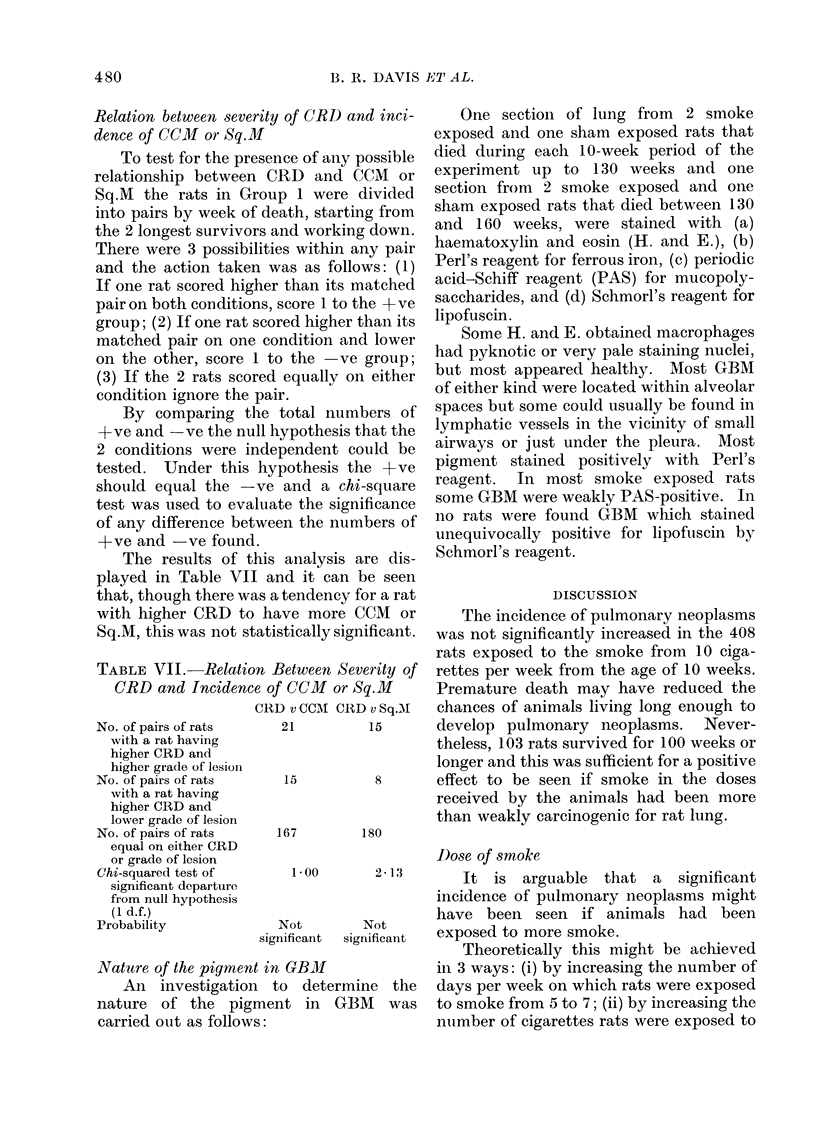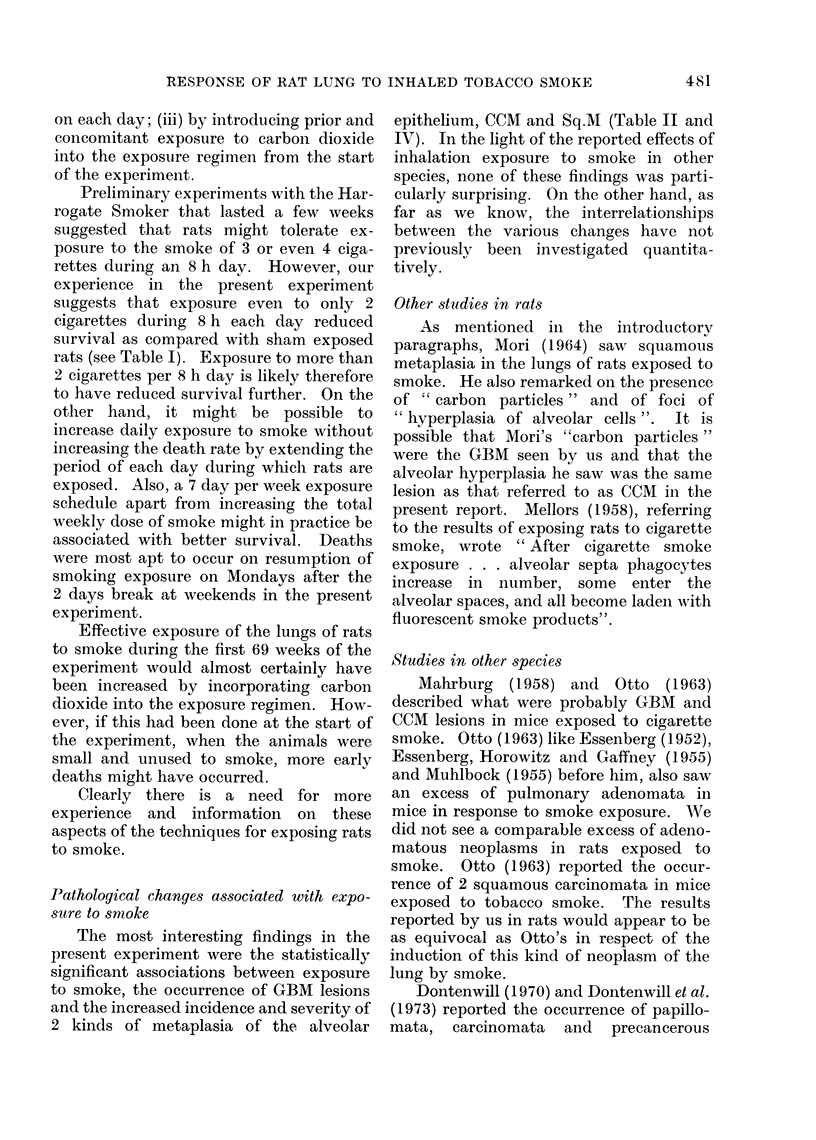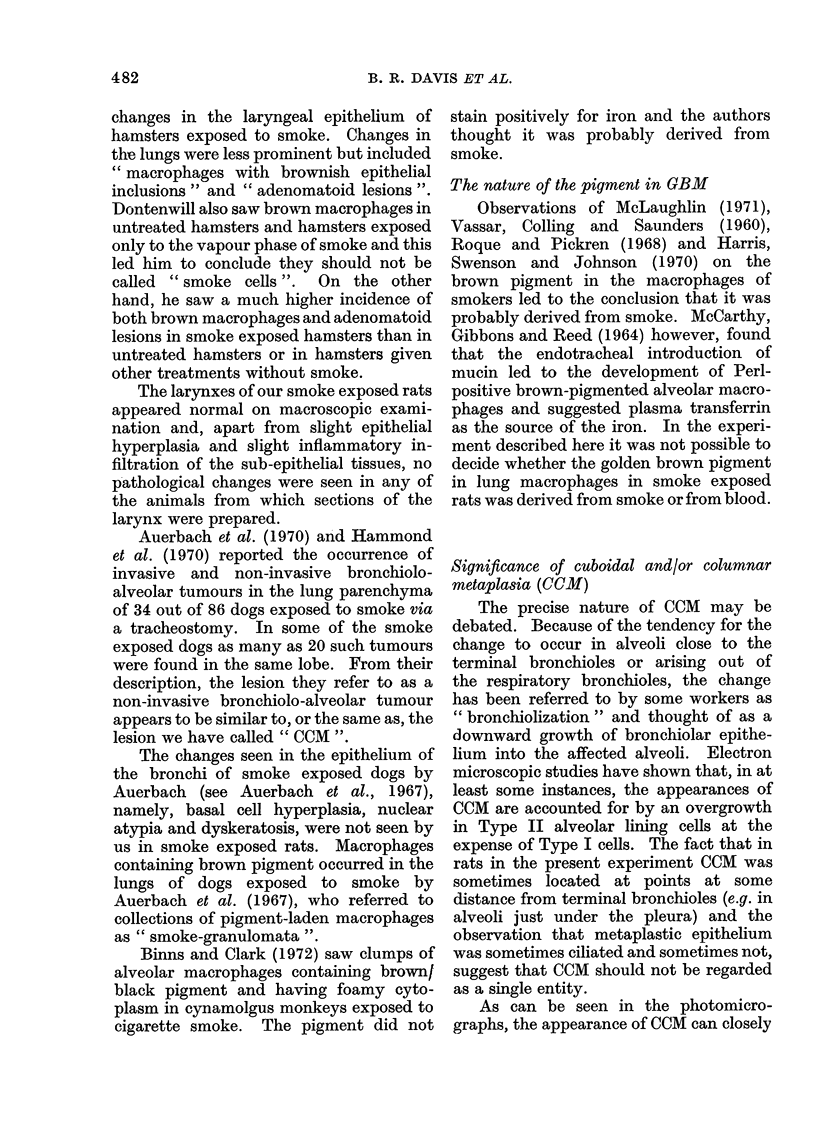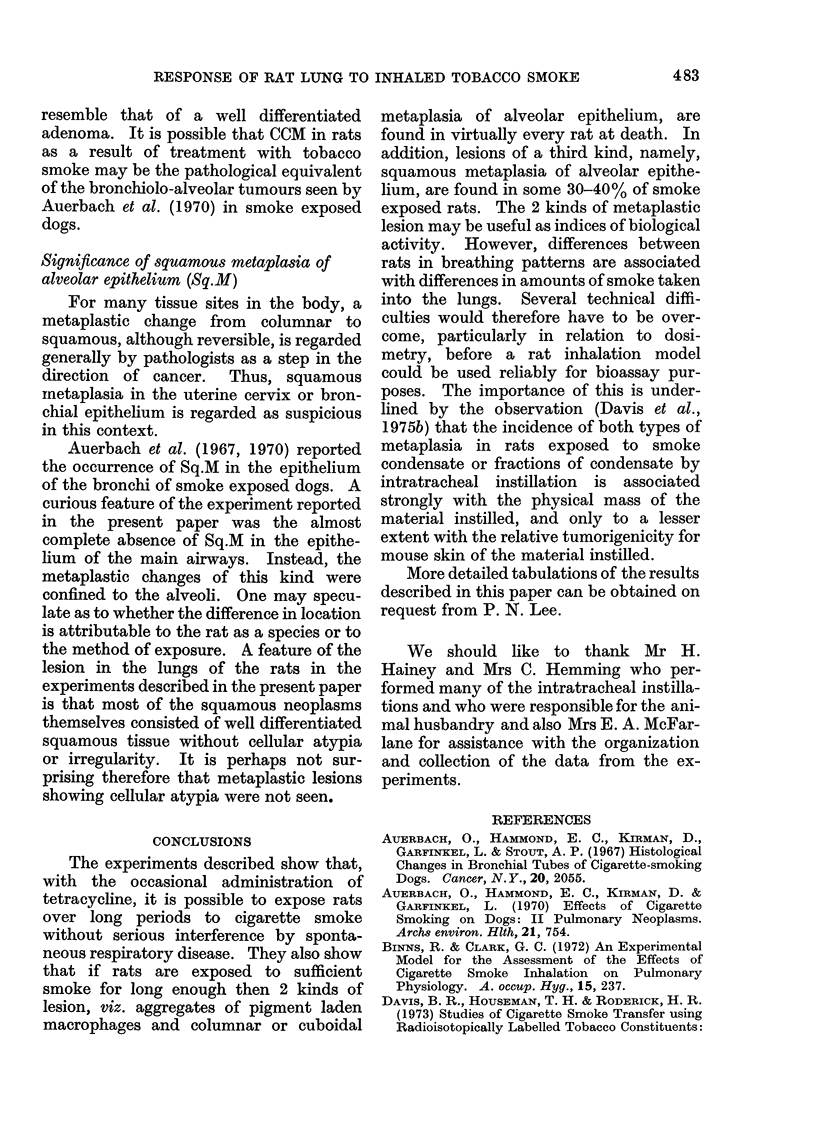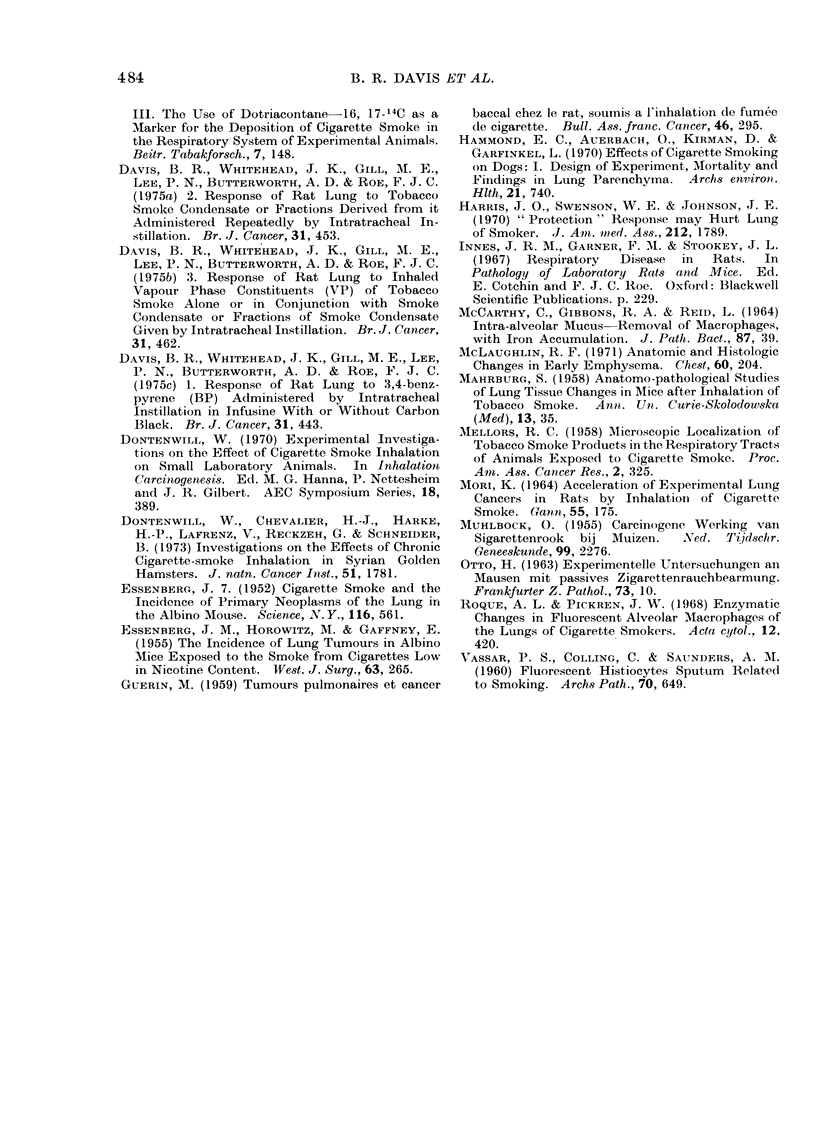# Response of rat lung to inhaled tobacco smoke with or without prior exposure to 3,4-benzpyrene (BP) given by intratracheal instillation.

**DOI:** 10.1038/bjc.1975.87

**Published:** 1975-04

**Authors:** B. R. Davis, J. K. Whitehead, M. E. Gill, P. N. Lee, A. D. Butterworth, F. J. Roe

## Abstract

**Images:**


*Br. J. Cancer* (1975) **31**, 469

### RESPONSE OF RAT LUNG TO INHALED TOBACCO SMOKE WITH OR WITHOUT PRIOR EXPOSURE TO 3,4-BENZPYRENE (BP) GIVEN BY INTRATRACHEAL INSTILLATION

B. R. DAVIS, J. K. WHITEHEAD, M. E. GILL, P. N. LEE, A. D. BUTTERWORTH AND F. J. C. ROE

*From the Tobacco Research Council Laboratories, Otley Road, Harrogate*

Received 27 August 1974. Accepted 2 January 1975

**Summary.—SPF rats were exposed to the smoke from 10 cigarettes per week from the age of 10 weeks until they died. Survival, body weight, tumour incidence and histopathological appearances of the lungs were compared with those for untreated sham exposed rats. Two further groups were given a single dose of 3,4-benzyprene (BP) by intratracheal instillation. One of these was then exposed to the smoke of 10 cigaretes per week till death.**

**Compared with untreated or sham exposed rats, exposure to smoke was associated with a significant reduction in incidence of mammary tumours.**

**Exposure to smoke was associated with an increasing incidence of collections of macrophages laden with golden-brown pigment (GBM) and of areas of cuboidal or columnar metaplasia (CCM) or squamous metaplasia (Sq.M) of alveolar epithelium. In control rats there was virtually no GBM, a low incidence of CCM and Sq.M. Four out of 406 smoke exposed rats which came to post mortem had squamous neoplasms in the lungs, 3 having lesions of doubtful malignancy and one having a squamous carcinoma. In contrast, no squamous neoplasms were seen in 197 control rats. This difference was not statistically significant.**

**The findings in rats given a single dose of BP were, in all the above respects, similar to those in untreated rats, except that one developed a squamous carcinoma of the lung. The effects of a single dose of BP followed by smoke exposure were in general similar to those of smoke exposure only. Three rats on this treatment regimen developed squamous cancers of the lung. None of the treatments increased the incidence of adenomata of the lungs.**

**The results are discussed in relation to other studies of the effects of smoke exposure on rats and other species.**

AN APPARATUS, known as the Harrogate Smoker, for exposing rats to fresh tobacco smoke by inhalation has been described elsewhere (Davis, Houseman and Roderick, 1973). The main objective of the experiment described in the present paper was to see whether squamous cancers of the lungs could be induced by the repeated exposure of rats to tobacco smoke throughout their life-span using the Harrogate Smoker. A subsidiary objective was to see whether squamous cancer would arise in response to a single intratracheal dose of benzo(a)pyrene followed by life-long exposure.

Relatively few previous studies of the effects of long-term exposure of rats to inhaled tobacco smoke have been reported. Mori (1964) saw pulmonary adenomata in 2, and areas of squamous metaplasia in 3, out of 14 rats exposed to cigarette smoke by inhalation for up to 186 days. Guerin (1959) reported the occurrence of 3 malignant and 2 benign neoplasms of the epithelium of the mouth among 68 rats exposed in a chamber to cigarette smoke

For the author's present address and address for reprints, see p. 443

by inhalation for up to 2 years. Three of the 68 rats, compared with 1 out of 40 controls, developed adenomatous tumours of the lung. One smoke exposed rat developed a small metastasizing squamous carcinoma of the lung. Guerin remarked on the absence of squamous metaplasia of the lungs of the rats in his study.

### MATERIALS AND METHODS

*Rats.*—A total of 780 female non-inbred Wistar specified pathogen-free (SPF) rats were obtained from Scientific Products Limited. They were allocated by a non-selective process to the 5 groups shown in Table I. The rats were aged approximately 7 weeks on arrival in the Experimental Unit and were kept there for 3 weeks without treatment. Details of diet, caging and periodic treatment with tetracycline to counter nonspecific respiratory disease are given in a parallel paper (Davis *et al.*, 1975*c*).

*Chemicals.*—For specifications of 3,4-benzypyrene (BP), carbon black (CB) and infusine (I) see Davis *et al.* (1975*c*).

*Cigarettes.*—For specification of the cigarettes (T29) used, see Davis *et al.* (1975*a*).

*Preparation of suspension of BP in I + CB for intratracheal instillation and technique of intracheal instillation.*—For details see Davis *et al.* (1975*c*).

*Exposure of rats to tobacco smoke in the "Harrogate Smoker".*—A full description of the "Harrogate Smoker" apparatus is given by Davis *et al.* (1973). For the experiment now described, the apparatus was adjusted to take one puff of 25 ml vol and 2 sec duration regularly once every min into a chamber containing 100 ml air and to hold the 1 in 5 smoke air mixture in the chamber for a period of 15 sec. Rats were fitted snugly into Perspex tubes such that their noses protruded into a smoke chamber. When in position rats were offered fresh air to breathe during 45 sec of each min and a 1 in 5 fresh smoke/air mixture during 15 sec of each min. Rats were thus exposed to the smoke of one cigarette twice each day, once in the morning and once in the afternoon, on 5 days of each week for the whole of their lives from the age of 10 weeks. Eleven puffs of 25 ml each were taken from each cigarette. The unsmoked butt after 11 puffs averaged 20 mm.

After the experiment had been in progress for about a year, the question arose whether rats were able to hold their breath throughout the 15 sec of the cycle during which the smoke/air mixture was offered to them and thereby avoid exposure to smoke. Simple observation suggested that some animals stopped breathing for at least a short time when smoke came into the chamber. To overcome doubts on this score, after a number of preliminary tests it was decided to introduce exposure to $CO_2$ into the exposure regimen. For 5 min before the start of each smoke exposure session, rats were given only a 95% air: 5% $CO_2$ mixture to breathe. During smoke exposure a mixture of the same composition was offered to rats instead of air during the 45 sec of the cycle when no smoke was in the chamber. (The atmosphere in which the cigarette burnt down was, of course, air and not the air: $CO_2$ mixture.)

In the preliminary tests involving 20 rats, it was found that rats would tolerate exposure to smoke and to $CO_2$ in the way described and that inclusion of $CO_2$ in the exposure regimen increased the mean carboxyhaemoglobin levels after exposure to the smoke from one cigarette (8·45% ± 1·00% standard error (s.e.) compared with 3·90% ± 0·44% s.e.).

In another experiment, further evidence of improved breathing with $CO_2$ stimulation was obtained using cigarettes impregnated with $^{74}$As (as arsenious sulphate). Although not an ideal particulate phase marker (since small amounts of radioactive arsine were detected in the vapour phase), the use of $^{74}$As did give an indication of total smoke inhaled and retained by exposed rats. The respective mean amounts of radioactivity recovered from the lungs of 10 $CO_2$ stimulated and 10 non-stimulated animals were 17·45% ± 1·71% and 8·46% ± 1·24% s.e. of the mean total radioactivity transferred to the exposure chamber. These means were significantly different at the $P < 0·001$ level.

Exposure to carbon dioxide for 5 min before and during each smoking session was introduced during the 69th week of the experiment.

*Distribution of inhaled smoke particulate matter.*—Recently, dotriacontane-16, 17-$^{14}$C has been used by Davis *et al.* (1973) as a marker for the deposition of particulate matter within the respiratory tract of animals exposed to smoke in the Harrogate

Smoker. They found that in 20 rats each exposed to the smoke from one labelled cigarette without $CO_2$ stimulation an average of 2·2% ± 0·7% s.e. of the mainstream particulate matter was deposited in the head and an average of 7·0% ±1·5% in the larynx, trachea and lungs. This ratio of head : lung deposition is different from that reported for hamsters and for mice exposed to smoke by other exposure devices. Greater deposition of smoke particulate matter in the lungs than in the head has also been seen in recent experiments (P. J. Simons, unpublished) in which rats were exposed to various dilutions of smoke in an apparatus similar to the Harrogate Smoker. The actual ratios appeared to depend on the breathing patterns of individual animals.

*Observations made during experiments.*—Animals were examined every day, including Saturdays and Sundays, for state of general health. Sick animals thought to be moribund were killed with chloroform immediately before post-mortem examination.

During the later stages of the experiment, some animals developed subcutaneous tumours which were presumed to be of mammary origin. If animals bearing such tumours appeared to be otherwise healthy, the tumours were, in some cases, removed surgically under ether anaesthesia.

Animals were weighed individually at not less than 4-week intervals.

*Post-mortem procedure.*—See Davis *et al.* (1975*c*).

*Microscopic examination of tissues.*—In the first instance all the sections derived from animals in the experiment were examined, and the results recorded as described in Davis *et al.* (1975*c*).

As explained below, the slides prepared from the lungs of animals exposed to smoke were re-examined " blind " to ascertain the relation between length of exposure to smoke and incidence and severity of particular lung changes.

Details of grading systems used for chronic respiratory disease (CRD), columnar and cuboidal metaplasia of alveolar epithelium (CCM), squamous metaplasia (Sq.M) and squamous neoplasia (Sq.N) are also given in Davis *et al.* (1975*c*).

A feature of the effects of cigarette smoke by inhalation was the aggregation in the lungs of macrophages containing a characteristic golden-brown coloured pigment (GBM) (See Fig. 2, 3). Four grades were used to quantify the size and numbers of these macrophages and 2 slides from each smoke-exposed rat were graded as follows: Grade 0: none; Grade 1: isolated small aggregations (affecting only 1 or 2 adjacent alveoli); Grade 2: moderately numerous aggregations; and Grade 3: numerous aggregations seen in every low-power field examined.

*Statistical methods.*—See Davis *et al.* (1975*c*).

*Incidence of various lung lesions in rats dying at different times during the experiment and interrelationship between various lung lesions.*—The main histopathological evaluation of the lung sections was carried out by one pathologist (B.R.D.) and the results used to compare the incidence and severity of the various lesions between the different treatment groups. It was also of interest to examine how time of death in relation to the start of treatment influenced the incidence and severity of GBM, CCM and Sq.M and to see whether there were interrelationships between these lesions. For this, attention was restricted to those animals exposed to smoke (*i.e.* Group 1), and a second " blind " examination was carried out by a second pathologist (F.J.C.R.). In the case of CRD the 2 pathologists used different criteria: B.R.D. classified terminal bronchopneumonia as CRD Grade 3 whereas F.J.C.R. either regarded such rats as ungradable or assessed the severity of CRD on the basis of appearances in lung lobes or lobules not involved in the bronchopneumonic process.

To investigate the interrelationships between GBM, CCM and Sq.M, the CCM grade used was the mean of grades (estimated by F.J.C.R.) on 2 or more lung sections. The grades for Sq.M used in the same investigation were based on an assessment of the incidence and severity of the lesion in all available lung sections for each animal.

### RESULTS

Differences were observed between groups in survival, in body weight change and in the incidence of certain pathological changes in the lungs. The incidence of neoplasms arising in sites other than the lung was similar in all 5 groups, except that there was a significantly lower incidence of mammary tumours in rats of the 2 groups exposed to tobacco smoke. The findings are summarized and illustrated in Tables I–III and Fig. 1–5.

Pathological changes occurring in the lungs were of 2 kinds; those seen in approximately the same incidence in all groups (*e.g.* CRD) and those seen frequently in smoke exposed rats but only uncommonly or never in rats of other groups (*e.g.* GBM, CCM and Sq.M). The concordance between these latter changes and the relationship between their incidence and length of exposure or age are shown in Tables IV and V.

*Effect of exposure to tobacco smoke on survival (Table I)*

Rats that received no treatment during the first 20 weeks of the experiment but which were thereafter put in a smoke exposure tube twice a day on 5 days per week without being actually exposed to smoke (Group 5) survived almost as well (mean survival time = 109 weeks) as the

TABLE I.—*Effect of Treatment on Survival*

| Group | Treatment | Age at first treatment (weeks) | No. of rats alive at end of treatment week | | | | | | | | | Mean survival from start of treatment (weeks) |
|---|---|---|---|---|---|---|---|---|---|---|---|---|
| | | | 0 | 20 | 40 | 60 | 80 | 100 | 120 | 140 | 160 | |
| 1 | Exposed to the smoke from 1 T29 cigarette × 10 weekly for life | 10 | 408 | 333 | 258 | 186 | 143 | 103 | 61 | 0 | — | 63 |
| 2 | 2 mg 3,4 benzpyrene (BP) with infusine (I) and carbon black (CB) by intratracheal instillation once only | 10 | 84 | 84 | 83 | 79 | 71 | 56 | 35 | 7 | 0 | 108 |
| 3 | As group 2 then smoke from 1 T29 cigarette × 10 weekly for life | 10 | 84 | 64 | 53 | 40 | 37 | 24 | 12 | 4 | 0 | 65 |
| 4 | Untreated throughout life | 10 | 102 | 102 | 102 | 100 | 93 | 74 | 43 | 4 | 0 | 113 |
| 5 | Untreated for first 20 weeks of the experiment and thereafter sham exposed to smoke (*i.e.* put in a tube on the Harrogate Smoker × 10 weekly but not exposed to smoke) | 10 | 102 | 102 | 101 | 97 | 91 | 67 | 35 | 6 | 0 | 109 |

TABLE II.—*Effect of Treatment on Incidence of Squamous Neoplasms (Sq.N) and on Mean Grades of Severity of Chronic Respiratory Diseases (CRD), and of Columnar and Cuboidal Metaplasia (CCM) and Squamous Metaplasia (Sq.M) of Alveolar Epithelium**

| Group | Treatment | Mean grade | | | Squamous neoplasms | | | |
|---|---|---|---|---|---|---|---|---|
| | | CRD | CCM | Sq.M | Grade 4 | Grade 5 | Grade 6 | Grade All |
| 1 | Smoke from 1 cigarette × 10 weekly | 1·88 (1·81)$^{+}$ | 0·97 (0·53)$^{+++}$ | 0·31 (0·19)$^{+++}$ | 3 | 1 | 0 | 4 (4·4) |
| 2 | 2 mg BP in I+CB once | 1·94 (2·10)$^{-}$ | 0·25 (1·02)$^{---}$ | 0·10 (0·30)$^{-}$ | 0 | 0 | 1 | 1 (1·0) |
| 3 | 2 mg BP in I+CB then smoke from 1 cigarette × 10 weekly | 1·89 (1·80) | 0·66 (0·53) | 0·27 (0·17) | 0 | 2 | 1 | 3 (0·8)$^{+}$ |
| 4 | Untreated | 1·92 (2·08)$^{-}$ | 0·32 (1·06)$^{---}$ | 0·09 (0·28)$^{-}$ | 0 | 0 | 0 | 0 (0·7) |
| 5 | Untreated for 20 weeks then sham smoke exposed | 1·97 (2·06) | 0·35 (0·94)$^{---}$ | 0·03 (0·26)$^{--}$ | 0 | 0 | 0 | 0 (1·1) |

* The numbers in parentheses are those expected as calculated by the method described in the text (see p. 445). Significance is indicated as follows: +, ++ and +++ show that O exceeded E with probabilities of $P < 0 \cdot 05$, $P < 0 \cdot 01$, and $P < 0 \cdot 001$ respectively and −, −− and −−− show that E exceeded O with probabilities of $P < 0 \cdot 05$, $P < 0 \cdot 01$ and $P < 0 \cdot 001$ respectively.

FIG. 2.—Lung from rat that came to post mortem 21 weeks after the start of exposure to 10 cigarettes per week in the Harrogate Smoker. (Rat No. 348/5, Group No. 1). The photomicrograph shows golden brown pigment-containing macrophages associated with cuboidal metaplasia in which many of the cells are ciliated. H. and E. × 265.

FIG. 3.—Lung from rat that came to post mortem 112 weeks after the start of exposure to 10 cigarettes/ week in the Harrogate Smoker. (Rat No. 346/4, Group No. 1.) The photomicrograph shows squamous metaplasia in the alveolar epithelium with some keratinization. Golden brown pigment-laden macrophages were present in large numbers in the lungs of this rat and some are visible in the picture. H. and E. × 105.

FIG. 4.—Lung from rat that came to post-mortem 72 weeks after the start of exposure to 10 cigarettes/ week in the Harrogate Smoker. (Rat No. 349/5, Group 1.) The photomicrograph shows the almost complete replacement of the lobe by a squamous carcinoma. H. and E. × 40.

FIG. 5.—Higher power view of lung shown in Fig. 4. The squamous nature of the tumour and its active invasion of the surrounding tissues are clearly visible. H. and E. × 215.

TABLE III.—*Incidence of Extrapulmonary Neoplasms**

| Group | Treatment | Mammary tumours removed during the experiment | Mammary tumours present at post mortem | Total mammary tumours† | Uterus | Ovary | Lympho-reticular | Con-nective tissue | Pituitary | Adrenal | Other | All except mammary |
|---|---|---|---|---|---|---|---|---|---|---|---|---|
| 1 | Smoke from 1 cigarette × 10 weekly | 8 | 29 | 37 (55·6)$^{--}$ | 0 (2·1) | 1 (4·2) | 8 (16·9) | 6 (4·5) | 5 (7·9) | 6 (4·2) | 6 (6·8) | 31 (44·1)$^{-}$ |
| 2 | 2 mg BP in I + CB once | 7 | 19 | 26 (21·6) | 1 (0·9) | 4 (1·7) | 8 (6·6) | 2 (2·1) | 4 (3·3) | 2 (1·8) | 5 (3·4) | 26 (18·5)$^{+}$ |
| 3 | 2 mg BP in I + CB then smoke from 1 cigarette × 10 weekly | 1 | 2 | 3 (12·0)$^{--}$ | 1 (0·5) | 1 (1·2) | 6 (3·9) | 0 (1·1) | 1 (2·3) | 0 (0·8) | 0 (1·4) | 8 (10·6) |
| 4 | Untreated | 5 | 37 | 42 (29·8)$^{++}$ | 2 (1·2) | 1 (2·3) | 14 (9·5) | 1 (3·1) | 4 (4·7) | 1 (2·2) | 5 (4·6) | 28 (25·8) |
| 5 | Untreated for 20 weeks then sham exposed | 10 | 30 | 40 (29·0)$^{+}$ | 2 (1·4) | 5 (2·5) | 11 (10·1) | 5 (3·3) | 9 (4·8)$^{+}$ | 2 (2·0) | 5 (4·7) | 33 (27·0) |

* Expected values based on incidence in all the 763 rats of the present experiment which were examined post mortem on the assumption that treatment had no effect on incidence. Significance levels indicated as in Tables II–IV.

† In the calculation of the " expected " values for mammary tumours, the animal was assumed to have died at the date the tumour was removed.

TABLE IV.—*Relation Between Length of Exposure to Smoke and Incidence of GBM, CCM and Sq.M in Mice of Group* 1

| Period (weeks) | No. dying with good lung slides | GBM mean grade | GBM % grade>O | CCM mean grade | CCM % grade>O | Sq.M mean grade | Sq.M % grade>O |
|---|---|---|---|---|---|---|---|
| 0–9 | 46 | 0·10 | 13·0 | 0·10 | 8·7 | 0·00 | 0·00 |
| 10–19 | 24 | 0·52 | 62·5 | 0·10 | 12·5 | 0·04 | 4·17 |
| 20–29 | 43 | 1·33 | 95·3 | 0·37 | 44·2 | 0·12 | 4·65 |
| 30–39 | 35 | 1·77 | 97·1 | 0·34 | 37·1 | 0·00 | 0·00 |
| 40–49 | 38 | 1·92 | 100·0 | 0·43 | 42·1 | 0·05 | 5·27 |
| 50–59 | 21 | 2·12 | 100·0 | 0·62 | 61·9 | 0·10 | 4·76 |
| 60–69 | 18 | 2·39 | 100·0 | 0·83 | 66·7 | 0·06 | 5·56 |
| 70–79 | 24 | 2·46 | 100·0 | 1·38 | 95·8 | 0·58 | 29·17 |
| 80–89 | 22 | 2·64 | 100·0 | 1·07 | 90·9 | 0·41 | 22·73 |
| 90–99 | 17 | 2·50 | 100·0 | 1·18 | 94·1 | 0·41 | 29·41 |
| 100–109 | 21 | 2·74 | 100·0 | 1·91 | 100 | 0·43 | 28·57 |
| 110–119 | 22 | 2·61 | 100·0 | 1·75 | 95·5 | 0·32 | 18·18 |
| 120–129 | 33 | 2·88 | 100·0 | 2·00 | 100 | 0·82 | 39·39 |
| 130–160 | 27 | 2·63 | 100·0 | 1·89 | 100 | 0·78 | 37·04 |

FIG. 1.—Effect of treatment on body weight.

untreated group (Group 4: mean survival time = 113 weeks). Exposure to the smoke of 1 cigarette twice daily on 5 days per week (Group 1), however, reduced the mean survival time to 63 weeks. Rats given a single dose of 2 mg BP in I + CB (Group 2) survived (mean survival time = 108 weeks) as well as the untreated rats, but similar treatment with BP in I + CB followed by twice daily smoke exposure (Group 3) reduced the mean survival time to 65 weeks.

*Effect of exposure to tobacco smoke on body weight* (*Fig.* 1)

A single dose of BP in I + CB marginally ($P \simeq 0{\cdot}1$) reduced body growth compared with that of untreated rats. Sham

TABLE V.—*Relationship Between Sq.M and the Other Lesions*

| Sq.M grade | No. of rats | Excess CCM | | Excess GBM | |
|---|---|---|---|---|---|
| | | Mean | S.E. | Mean | S.E. |
| 0 | 349 | 0·0 | | 0·0 | |
| 1 | 30 | +0·40 | 0·16 | +0·34 | 0·11 |
| 2 | 11 | +1·12 | 0·32 | +0·19 | 0·18 |
| 3 | 12 | +1·08 | 0·18 | +0·11 | 0·20 |
| 4 | 3 | −0·33 | 0·22 | −0·30 | 0·39 |
| 5 | 1 | −0·59 | — | −0·46 | — |
| All Sq.M | 57 | +0·63 | 0·12 | +0·22 | 0·08 |

exposure had a more marked effect ($P < 0{\cdot}01$) and twice daily exposure to smoke greatly reduced weight gain ($P < 0{\cdot}0001$). The effect of sham exposure can be clearly seen in the drop in body weight between week 30, when it began, and week 34. The introduction of $CO_2$ into the exposure regimen for rats of Groups 1 and 3 at 69 weeks had no observed effect on body weight gain.

*Effect of exposure to tobacco smoke on incidence and severity of CRD (Table II)*

At post mortem all rats showed some evidence of nonspecific lung disease but in most cases the disease was not of more than minimal or moderate severity. Taking into account survival differences there was a slightly, but highly significantly ($P < 0{\cdot}001$), greater incidence of CRD in rats of the 2 smoke-exposed groups than in rats of the other groups.

In the assessment of the severity of CRD, the presence and severity of interstitial pneumonitis, of lymphocytic infiltration around main airways and of consolidation and fibrosis were taken into account. For the purposes of preparing Table II terminal bronchopneumonia was classified as grade 3 (*i.e.* severe CRD).

*Other pathological changes in the lungs*

A variety of other changes (*e.g.* focal collections of clear macrophages, foci of macrophages containing black pigment, ova of parasitic worms, granulomatous lesions and foreign bodies (see Innes, Garner and Stookey, 1967)) were seen in the lungs of rats of all groups and no attempt was made to quantify them.

No changes were seen in the epithelium of the main airways other than slight basal cell hyperplasia, slight crowding of epithelial cells and folding of the mucosal layers and slight increase in goblet cells in some rats. These changes were more frequent in the smoke exposed animals of Groups 1 and 3 than in rats in the groups that were not exposed to smoke.

*Incidence of columnar and cuboidal metaplasia of alveolar epithelium (CCM) (Table II and Fig. 2)*

The incidence of CCM in rats exposed to cigarette smoke (Groups 1 and 3) was significantly higher than that in rats in the other 3 groups.

*Incidence of alveolar squamous metaplasia Sq.M) and squamous neoplasms (Sq.N) of the lung (Table II and Fig. 3–5)*

Six out of 96 untreated rats (Group 4) and 1 out of 106 sham exposed rats (Group 5) showed areas of Sq.M in the lungs, mostly of grade 1 severity. By contrast, 55 out of 406 smoke exposed rats (Group 1) showed squamous lesions, 50 of these being grade 2 or more. Three rats in this group had squamous neoplasms of doubtful malignancy and one had a locally invasive squamous carcinoma. The excess incidence of all squamous lesions in Group 1 was highly significant as compared with Groups 4 or 5 ($P < 0{\cdot}001$). However, the incidence of squamous neoplasms only was not significantly greater in Group 1 than in the control groups.

One rat of Group 2 (2 mg BP in I + CB and no further treatment) had, when it died, a squamous carcinoma of the lung extending beyond the lobe of origin, but the incidence of Sq.M in this group was lower, but not significantly so, than that in untreated rats. Of 83 rats given 2 mg BP in 1 + CB once, followed by smoke exposure (Group 3), 3 developed squamous carcinomata and a further 5 had grade 1 or grade 2 Sq.M.

If one assumes that the squamous lesions designated as grades 0–6 constitute a natural biological series and that the numerical designations of grades reflect their relative severity *i.e.* that 1 rat with a grade 6 lesion is equivalent to 6 rats with grade 1 lesions), then the 2 groups exposed to tobacco smoke (Groups 1 and 3) do not differ significantly from each other, but together have a mean grade that is significantly higher than that in the non smoke exposed Groups 2, 4 and 5 ($P < 0{\cdot}001$).

It is noteworthy that, if attention is restricted to Sq.N, then 7 out of 489 smoke exposed rats had this lesion compared with only 1 out of 274 rats that were not exposed to smoke. These proportions do not differ statistically. Surprisingly, there was no evidence of any trend of increasing incidence of Sq.N with age so that for this lesion, unlike the others, standardization for age of death, did not make any real difference. In fact, of the 7 Sq.N observed in the 2 smoke exposed groups, 6 occurred in the 341 animals dying in the first 90 weeks of the experiment and only 1 in the 146 animals dying subsequently.

*Incidence of pulmonary adenomata*

Six pulmonary adenomata were observed, 4 in 489 smoke exposed rats and 2 in 274 rats in the other groups. There was no evidence, therefore, that any of the treatments affected the incidence of neoplasms of this type.

*Incidence of extrapulmonary neoplasms*

As indicated above (p. 472), it proved necessary to excise certain subcutaneous tumours. Most were neoplasms of mammary gland origin. A few consisted of areas of cystic mastitis.

The incidence of surgically removed mammary tumours and of extrapulmonary tumours of various sites present at death is shown in Table III, together with the incidence of each kind of neoplasm expected in the light of survival experience and on the assumption that treatment had no effect.

Rats in the two groups exposed to tobacco smoke (Groups 1 and 3) developed significantly fewer mammary tumours than rats in the other groups ($P < 0{\cdot}001$). In this respect, sham exposed rats were similar to untreated animals.

One hundred and forty-eight rats developed one or more mammary tumours. Most of the tumours were benign fibroadenomata or adenomata with or without evidence of secretory activity. Some benign intraduct papillomata were also encountered. Only 10 rats had mammary tumours which were regarded as malignant, *i.e.* adenocarcinomata. The numbers of rats that had malignant mammary tumours in relation to the number examined postmortem were Group 1—2/406, Group 2—2/77, Group 3—0/83, Group 4—2/96 and Group 5—4/101. The lower incidence in Groups 1 and 3 is partly attributable to the poorer survival of rats in these groups. After correction for survival, none of the observed values differed significantly from the expected ones.

Two other differences significant at the $P < 0{\cdot}05$ level were an excess (O = 9, E = 4·8) of chromophobe adenomata of the pituitary gland in the sham exposed group and a deficiency (O = 8, E = 16·9) of neoplasms of lymphoreticular tissues in the rats exposed only to tobacco smoke. Since 40 comparisons of " observed " and " expected " were made in this analysis (5 groups × 8 sites), it is not surprising that results significant at this level were thrown up occasionally. Insofar as the deficiency of lymphoreticular neoplasms in Group 1 was not matched by a similar deficiency in the group given BP in I + CB before smoke exposure (Group 3), and the excess of pituitary tumours seen in sham exposed rats (Group 5) was not matched by any excess incidence in the 2 smoke exposed groups, it would be advisable to regard these differences as of doubtful significance unless confirmed by further work.

*Relation between length of exposure to smoke and incidence of GBM, CCM and Sq.M*

The results of the "blind" examination of lung slides from rats of Group 1 is shown in Table IV.

The mean grade for GBM rose rapidly after 10 weeks, GBM lesions being seen in almost all rats dying after 19 weeks. The last rat to die with no GBM lesion was at week 34. By 40 weeks, the mean grade had reached about 2 and only increased slowly thereafter. After week 101 every animal had a GBM grade of 2 or higher.

The mean CCM grade rose in a fairly continuous manner throughout the experiment. More than half of the rats dying during each 10-week period from 50 weeks onwards showed CCM and after 70 weeks more than 90% of rats had CCM lesions.

Sq.M was uncommon until about week 70, only about 1 rat in 20 that died before this time having the lesion. After that the rate jumped to 20% and remained that or more until the end of the experiment. One rat died after only 26 weeks with a grade 4 squamous lesion. Since it had no CCM and a GBM score of only 0·5, it seems unlikely that this lesion was caused by treatment.

Between the 60–69 and 70–79 week death groups the proportion of rats dying with CCM jumped from 66·7 to 95·8% and of rats dying with Sq.M from 5·6 to 29·1%. One naturally wonders whether the introduction of exposure to carbon dioxide during week 69 (see p. 472) was in any way responsible for these jumps in incidence.

*Interrelationships between the 3 types of lesion*

In order to consider the interrelationships between the 3 types of lesions, it was clearly necessary to standardize for the time of death. Two slightly differing methods were used.

Firstly, one wished to see whether rats with Sq.M had higher incidences of CCM or GBM than expected for all rats dying at that week. For each rat with Sq.M, the differences between its CCM and GBM grades and the average grades of rats dying with Sq.M = 0 during that time period were calculated. These differences were summed over all time periods and the results displayed in Table V.

There was highly significant evidence that rats with Sq.M grades 1, 2 or 3 had a greater CCM than expected. The 4 rats with squamous tumours had less CCM than expected but this was possibly an artefact caused by difficulty of determining CCM in the presence of a tumour.

There was also significant evidence for rats with Sq.M having a higher GBM than expected but this difference was not so marked. It was clearest for rats with Sq.M = 1. This could be explained to some degree by the fact that this relationship was more marked in rats dying early when Sq.M values were low. Later on all rats had high GBM so the relationship tended to disappear.

Secondly, one wished to see whether CCM was interrelated with GBM independently of time. The method used was similar to that for Sq.M, except that instead of computing differences in CCM or GBM of rats with Sq.M > 0 compared with rats with Sq.M = 0, we computed differences in GBM of rats from the average GBM for that week and summed by CCM. The results displayed in Table VI indicate that there is a significant excess in GBM grade above expected as CCM increases.

TABLE VI.—*Relationship Between CCM and GBM*

| CCM grade | No. of rats | Difference in GBM from average: Mean | S.E. |
|---|---|---|---|
| 0·0 | 150 | −0·13 | 0·05 |
| 0·5 | 55 | −0·06 | 0·08 |
| 1·0 | 69 | +0·02 | 0·06 |
| 1·5 | 34 | +0·04 | 0·12 |
| 2·0 | 39 | +0·34 | 0·08 |
| 2·5 | 22 | +0·06 | 0·12 |
| 3·0 | 15 | +0·19 | 0·15 |
| 3·5 | 4 | +0·31 | 0·16 |
| 4·0 | 3 | +0·54 | 0·33 |

*Relation between severity of CRD and incidence of CCM or Sq.M*

To test for the presence of any possible relationship between CRD and CCM or Sq.M the rats in Group 1 were divided into pairs by week of death, starting from the 2 longest survivors and working down. There were 3 possibilities within any pair and the action taken was as follows: (1) If one rat scored higher than its matched pair on both conditions, score 1 to the +ve group; (2) If one rat scored higher than its matched pair on one condition and lower on the other, score 1 to the −ve group; (3) If the 2 rats scored equally on either condition ignore the pair.

By comparing the total numbers of +ve and −ve the null hypothesis that the 2 conditions were independent could be tested. Under this hypothesis the +ve should equal the −ve and a *chi*-square test was used to evaluate the significance of any difference between the numbers of +ve and −ve found.

The results of this analysis are displayed in Table VII and it can be seen that, though there was a tendency for a rat with higher CRD to have more CCM or Sq.M, this was not statistically significant.

TABLE VII.—*Relation Between Severity of CRD and Incidence of CCM or Sq.M*

| | CRD *v* CCM | CRD *v* Sq.M |
|---|---|---|
| No. of pairs of rats with a rat having higher CRD and higher grade of lesion | 21 | 15 |
| No. of pairs of rats with a rat having higher CRD and lower grade of lesion | 15 | 8 |
| No. of pairs of rats equal on either CRD or grade of lesion | 167 | 180 |
| *Chi*-squared test of significant departure from null hypothesis (1 d.f.) | 1·00 | 2·13 |
| Probability | Not significant | Not significant |

*Nature of the pigment in GBM*

An investigation to determine the nature of the pigment in GBM was carried out as follows:

One section of lung from 2 smoke exposed and one sham exposed rats that died during each 10-week period of the experiment up to 130 weeks and one section from 2 smoke exposed and one sham exposed rats that died between 130 and 160 weeks, were stained with (a) haematoxylin and eosin (H. and E.), (b) Perl's reagent for ferrous iron, (c) periodic acid–Schiff reagent (PAS) for mucopolysaccharides, and (d) Schmorl's reagent for lipofuscin.

Some H. and E. obtained macrophages had pyknotic or very pale staining nuclei, but most appeared healthy. Most GBM of either kind were located within alveolar spaces but some could usually be found in lymphatic vessels in the vicinity of small airways or just under the pleura. Most pigment stained positively with Perl's reagent. In most smoke exposed rats some GBM were weakly PAS-positive. In no rats were found GBM which stained unequivocally positive for lipofuscin by Schmorl's reagent.

### DISCUSSION

The incidence of pulmonary neoplasms was not significantly increased in the 408 rats exposed to the smoke from 10 cigarettes per week from the age of 10 weeks. Premature death may have reduced the chances of animals living long enough to develop pulmonary neoplasms. Nevertheless, 103 rats survived for 100 weeks or longer and this was sufficient for a positive effect to be seen if smoke in the doses received by the animals had been more than weakly carcinogenic for rat lung.

*Dose of smoke*

It is arguable that a significant incidence of pulmonary neoplasms might have been seen if animals had been exposed to more smoke.

Theoretically this might be achieved in 3 ways: (i) by increasing the number of days per week on which rats were exposed to smoke from 5 to 7; (ii) by increasing the number of cigarettes rats were exposed to on each day; (iii) by introducing prior and concomitant exposure to carbon dioxide into the exposure regimen from the start of the experiment.

Preliminary experiments with the Harrogate Smoker that lasted a few weeks suggested that rats might tolerate exposure to the smoke of 3 or even 4 cigarettes during an 8 h day. However, our experience in the present experiment suggests that exposure even to only 2 cigarettes during 8 h each day reduced survival as compared with sham exposed rats (see Table I). Exposure to more than 2 cigarettes per 8 h day is likely therefore to have reduced survival further. On the other hand, it might be possible to increase daily exposure to smoke without increasing the death rate by extending the period of each day during which rats are exposed. Also, a 7 day per week exposure schedule apart from increasing the total weekly dose of smoke might in practice be associated with better survival. Deaths were most apt to occur on resumption of smoking exposure on Mondays after the 2 days break at weekends in the present experiment.

Effective exposure of the lungs of rats to smoke during the first 69 weeks of the experiment would almost certainly have been increased by incorporating carbon dioxide into the exposure regimen. However, if this had been done at the start of the experiment, when the animals were small and unused to smoke, more early deaths might have occurred.

Clearly there is a need for more experience and information on these aspects of the techniques for exposing rats to smoke.

*Pathological changes associated with exposure to smoke*

The most interesting findings in the present experiment were the statistically significant associations between exposure to smoke, the occurrence of GBM lesions and the increased incidence and severity of 2 kinds of metaplasia of the alveolar epithelium, CCM and Sq.M (Table II and IV). In the light of the reported effects of inhalation exposure to smoke in other species, none of these findings was particularly surprising. On the other hand, as far as we know, the interrelationships between the various changes have not previously been investigated quantitatively.

*Other studies in rats*

As mentioned in the introductory paragraphs, Mori (1964) saw squamous metaplasia in the lungs of rats exposed to smoke. He also remarked on the presence of " carbon particles " and of foci of " hyperplasia of alveolar cells ". It is possible that Mori's "carbon particles " were the GBM seen by us and that the alveolar hyperplasia he saw was the same lesion as that referred to as CCM in the present report. Mellors (1958), referring to the results of exposing rats to cigarette smoke, wrote " After cigarette smoke exposure . . . alveolar septa phagocytes increase in number, some enter the alveolar spaces, and all become laden with fluorescent smoke products".

*Studies in other species*

Mahrburg (1958) and Otto (1963) described what were probably GBM and CCM lesions in mice exposed to cigarette smoke. Otto (1963) like Essenberg (1952), Essenberg, Horowitz and Gaffney (1955) and Muhlbock (1955) before him, also saw an excess of pulmonary adenomata in mice in response to smoke exposure. We did not see a comparable excess of adenomatous neoplasms in rats exposed to smoke. Otto (1963) reported the occurrence of 2 squamous carcinomata in mice exposed to tobacco smoke. The results reported by us in rats would appear to be as equivocal as Otto's in respect of the induction of this kind of neoplasm of the lung by smoke.

Dontenwill (1970) and Dontenwill *et al.* (1973) reported the occurrence of papillomata, carcinomata and precancerous changes in the laryngeal epithelium of hamsters exposed to smoke. Changes in the lungs were less prominent but included " macrophages with brownish epithelial inclusions " and " adenomatoid lesions ". Dontenwill also saw brown macrophages in untreated hamsters and hamsters exposed only to the vapour phase of smoke and this led him to conclude they should not be called " smoke cells ". On the other hand, he saw a much higher incidence of both brown macrophages and adenomatoid lesions in smoke exposed hamsters than in untreated hamsters or in hamsters given other treatments without smoke.

The larynxes of our smoke exposed rats appeared normal on macroscopic examination and, apart from slight epithelial hyperplasia and slight inflammatory infiltration of the sub-epithelial tissues, no pathological changes were seen in any of the animals from which sections of the larynx were prepared.

Auerbach *et al.* (1970) and Hammond *et al.* (1970) reported the occurrence of invasive and non-invasive bronchiolo-alveolar tumours in the lung parenchyma of 34 out of 86 dogs exposed to smoke *via* a tracheostomy. In some of the smoke exposed dogs as many as 20 such tumours were found in the same lobe. From their description, the lesion they refer to as a non-invasive bronchiolo-alveolar tumour appears to be similar to, or the same as, the lesion we have called " CCM ".

The changes seen in the epithelium of the bronchi of smoke exposed dogs by Auerbach (see Auerbach *et al.*, 1967), namely, basal cell hyperplasia, nuclear atypia and dyskeratosis, were not seen by us in smoke exposed rats. Macrophages containing brown pigment occurred in the lungs of dogs exposed to smoke by Auerbach *et al.* (1967), who referred to collections of pigment-laden macrophages as " smoke-granulomata ".

Binns and Clark (1972) saw clumps of alveolar macrophages containing brown/black pigment and having foamy cytoplasm in cynamolgus monkeys exposed to cigarette smoke. The pigment did not stain positively for iron and the authors thought it was probably derived from smoke.

*The nature of the pigment in GBM*

Observations of McLaughlin (1971), Vassar, Colling and Saunders (1960), Roque and Pickren (1968) and Harris, Swenson and Johnson (1970) on the brown pigment in the macrophages of smokers led to the conclusion that it was probably derived from smoke. McCarthy, Gibbons and Reed (1964) however, found that the endotracheal introduction of mucin led to the development of Perl-positive brown-pigmented alveolar macrophages and suggested plasma transferrin as the source of the iron. In the experiment described here it was not possible to decide whether the golden brown pigment in lung macrophages in smoke exposed rats was derived from smoke or from blood.

*Significance of cuboidal and/or columnar metaplasia (CCM)*

The precise nature of CCM may be debated. Because of the tendency for the change to occur in alveoli close to the terminal bronchioles or arising out of the respiratory bronchioles, the change has been referred to by some workers as " bronchiolization " and thought of as a downward growth of bronchiolar epithelium into the affected alveoli. Electron microscopic studies have shown that, in at least some instances, the appearances of CCM are accounted for by an overgrowth in Type II alveolar lining cells at the expense of Type I cells. The fact that in rats in the present experiment CCM was sometimes located at points at some distance from terminal bronchioles (*e.g.* in alveoli just under the pleura) and the observation that metaplastic epithelium was sometimes ciliated and sometimes not, suggest that CCM should not be regarded as a single entity.

As can be seen in the photomicrographs, the appearance of CCM can closely resemble that of a well differentiated adenoma. It is possible that CCM in rats as a result of treatment with tobacco smoke may be the pathological equivalent of the bronchiolo-alveolar tumours seen by Auerbach *et al.* (1970) in smoke exposed dogs.

*Significance of squamous metaplasia of alveolar epithelium (Sq.M)*

For many tissue sites in the body, a metaplastic change from columnar to squamous, although reversible, is regarded generally by pathologists as a step in the direction of cancer. Thus, squamous metaplasia in the uterine cervix or bronchial epithelium is regarded as suspicious in this context.

Auerbach *et al.* (1967, 1970) reported the occurrence of Sq.M in the epithelium of the bronchi of smoke exposed dogs. A curious feature of the experiment reported in the present paper was the almost complete absence of Sq.M in the epithelium of the main airways. Instead, the metaplastic changes of this kind were confined to the alveoli. One may speculate as to whether the difference in location is attributable to the rat as a species or to the method of exposure. A feature of the lesion in the lungs of the rats in the experiments described in the present paper is that most of the squamous neoplasms themselves consisted of well differentiated squamous tissue without cellular atypia or irregularity. It is perhaps not surprising therefore that metaplastic lesions showing cellular atypia were not seen.

### CONCLUSIONS

The experiments described show that, with the occasional administration of tetracycline, it is possible to expose rats over long periods to cigarette smoke without serious interference by spontaneous respiratory disease. They also show that if rats are exposed to sufficient smoke for long enough then 2 kinds of lesion, *viz.* aggregates of pigment laden macrophages and columnar or cuboidal metaplasia of alveolar epithelium, are found in virtually every rat at death. In addition, lesions of a third kind, namely, squamous metaplasia of alveolar epithelium, are found in some 30–40% of smoke exposed rats. The 2 kinds of metaplastic lesion may be useful as indices of biological activity. However, differences between rats in breathing patterns are associated with differences in amounts of smoke taken into the lungs. Several technical difficulties would therefore have to be overcome, particularly in relation to dosimetry, before a rat inhalation model could be used reliably for bioassay purposes. The importance of this is underlined by the observation (Davis *et al.*, 1975*b*) that the incidence of both types of metaplasia in rats exposed to smoke condensate or fractions of condensate by intratracheal instillation is associated strongly with the physical mass of the material instilled, and only to a lesser extent with the relative tumorigenicity for mouse skin of the material instilled.

More detailed tabulations of the results described in this paper can be obtained on request from P. N. Lee.

We should like to thank Mr H. Hainey and Mrs C. Hemming who performed many of the intratracheal instillations and who were responsible for the animal husbandry and also Mrs E. A. McFarlane for assistance with the organization and collection of the data from the experiments.

### REFERENCES

AUERBACH, O., HAMMOND, E. C., KIRMAN, D., GARFINKEL, L. & STOUT, A. P. (1967) Histological Changes in Bronchial Tubes of Cigarette-smoking Dogs. *Cancer, N.Y.*, **20**, 2055.

AUERBACH, O., HAMMOND, E. C., KIRMAN, D. & GARFINKEL, L. (1970) Effects of Cigarette Smoking on Dogs: II Pulmonary Neoplasms. *Archs environ. Hlth*, **21**, 754.

BINNS, R. & CLARK, G. C. (1972) An Experimental Model for the Assessment of the Effects of Cigarette Smoke Inhalation on Pulmonary Physiology. *A. occup. Hyg.*, **15**, 237.

DAVIS, B. R., HOUSEMAN, T. H. & RODERICK, H. R. (1973) Studies of Cigarette Smoke Transfer using Radioisotopically Labelled Tobacco Constituents:

III. The Use of Dotriacontane—16, 17-$^{14}$C as a Marker for the Deposition of Cigarette Smoke in the Respiratory System of Experimental Animals. *Beitr. Tabakforsch.*, **7**, 148.

DAVIS, B. R., WHITEHEAD, J. K., GILL, M. E., LEE, P. N., BUTTERWORTH, A. D. & ROE, F. J. C. (1975*a*) 2. Response of Rat Lung to Tobacco Smoke Condensate or Fractions Derived from it Administered Repeatedly by Intratracheal Instillation. *Br. J. Cancer*, **31**, 453.

DAVIS, B. R., WHITEHEAD, J. K., GILL, M. E., LEE, P. N., BUTTERWORTH, A. D. & ROE, F. J. C. (1975*b*) 3. Response of Rat Lung to Inhaled Vapour Phase Constituents (VP) of Tobacco Smoke Alone or in Conjunction with Smoke Condensate or Fractions of Smoke Condensate Given by Intratracheal Instillation. *Br. J. Cancer*, **31**, 462.

DAVIS, B. R., WHITEHEAD, J. K., GILL, M. E., LEE, P. N., BUTTERWORTH, A. D. & ROE, F. J. C. (1975*c*) 1. Response of Rat Lung to 3,4-benzpyrene (BP) Administered by Intratracheal Instillation in Infusine With or Without Carbon Black. *Br. J. Cancer*, **31**, 443.

DONTENWILL, W. (1970) Experimental Investigations on the Effect of Cigarette Smoke Inhalation on Small Laboratory Animals. In *Inhalation Carcinogenesis*. Ed. M. G. Hanna, P. Nettesheim and J. R. Gilbert. AEC Symposium Series, **18**, 389.

DONTENWILL, W., CHEVALIER, H.-J., HARKE, H.-P., LAFRENZ, V., RECKZEH, G. & SCHNEIDER, B. (1973) Investigations on the Effects of Chronic Cigarette-smoke Inhalation in Syrian Golden Hamsters. *J. natn. Cancer Inst.*, **51**, 1781.

ESSENBERG, J. 7. (1952) Cigarette Smoke and the Incidence of Primary Neoplasms of the Lung in the Albino Mouse. *Science, N.Y.*, **116**, 561.

ESSENBERG, J. M., HOROWITZ, M. & GAFFNEY, E. (1955) The Incidence of Lung Tumours in Albino Mice Exposed to the Smoke from Cigarettes Low in Nicotine Content. *West. J. Surg.*, **63**, 265.

GUERIN, M. (1959) Tumours pulmonaires et cancer baccal chez le rat, soumis a l'inhalation de fumée de cigarette. *Bull. Ass. franc. Cancer*, **46**, 295.

HAMMOND, E. C., AUERBACH, O., KIRMAN, D. & GARFINKEL, L. (1970) Effects of Cigarette Smoking on Dogs: I. Design of Experiment, Mortality and Findings in Lung Parenchyma. *Archs environ. Hlth*, **21**, 740.

HARRIS, J. O., SWENSON, W. E. & JOHNSON, J. E. (1970) " Protection " Response may Hurt Lung of Smoker. *J. Am. med. Ass.*, **212**, 1789.

INNES, J. R. M., GARNER, F. M. & STOOKEY, J. L. (1967) Respiratory Disease in Rats. In *Pathology of Laboratory Rats and Mice*. Ed. E. Cotchin and F. J. C. Roe. Oxford: Blackwell Scientific Publications. p. 229.

MCCARTHY, C., GIBBONS, R. A. & REID, L. (1964) Intra-alveolar Mucus—Removal of Macrophages, with Iron Accumulation. *J. Path. Bact.*, **87**, 39.

MCLAUGHLIN, R. F. (1971) Anatomic and Histologic Changes in Early Emphysema. *Chest*, **60**, 204.

MAHRBURG, S. (1958) Anatomo-pathological Studies of Lung Tissue Changes in Mice after Inhalation of Tobacco Smoke. *Ann. Un. Curie-Skolodowska (Med)*, **13**, 35.

MELLORS, R. C. (1958) Microscopic Localization of Tobacco Smoke Products in the Respiratory Tracts of Animals Exposed to Cigarette Smoke. *Proc. Am. Ass. Cancer Res.*, **2**, 325.

MORI, K. (1964) Acceleration of Experimental Lung Cancers in Rats by Inhalation of Cigarette Smoke. *Gann*, **55**, 175.

MUHLBOCK, O. (1955) Carcinogene Werking van Sigarettenrook bij Muizen. *Ned. Tijdschr. Geneeskunde*, **99**, 2276.

OTTO, H. (1963) Experimentelle Untersuchungen an Mausen mit passives Zigarettenrauchbearmung. *Frankfurter Z. Pathol.*, **73**, 10.

ROQUE, A. L. & PICKREN, J. W. (1968) Enzymatic Changes in Fluorescent Alveolar Macrophages of the Lungs of Cigarette Smokers. *Acta cytol.*, **12**, 420.

VASSAR, P. S., COLLING, C. & SAUNDERS, A. M. (1960) Fluorescent Histiocytes Sputum Related to Smoking. *Archs Path.*, **70**, 649.